# Supplementary material for: Working in labor and delivery unit increases the odds of work place violence in Amhara region referral hospitals: Cross-sectional study
Source: PLoS One. 2021 Oct 20;16(10):e0254962. doi: 10.1371/journal.pone.0254962 (PMC8528509; doi:10.1371/journal.pone.0254962)
Supplement: S1 File — (DOCX) [file pone.0254962.s001.docx]

**I: Information** **sheet to the participants**

**Research title**: Prevalence of workplace violence and associated factors among OBGYN department care providers of Amhara regional state referral hospitals Ethiopia 2019.

The main aim of this research project is to assess prevalence of workplace violence and associated factors among OBGYN department staffs of Amhara regional state referral hospitals Ethiopia 2019. It is helpful in designing and implementing conducive working environment while the health professionals on work place further to address quality care in serving mothers and their neonates.

**Risk of study:** study participant in this study will not have any risk.

**Right of study participants**: you have full right to participate or not to participate as well as you may end the interview after enrolled to participate at any time. You can ask any question you want.

**Confidentiality**: any information given from you will maintained confidential and we will not write personal identification such as name, phone cell number.

**Duration**: the survey will take usually about 20-30 minutes.

**Incentive /payment:** you will not have got any payment for participating in this study

This research project reviewed by Institutional Review Board (IRB) of University of Gondar, Department of Midwifery.

If you will have any concern you, can contact one of the concerned bodies and you may ask at any time you want.

You can contact to Eyaya Habtie Email: eyuhabt143@gmail.com Tel: +251943483822 and Mr. Tewodros Syoum Tel: +2519180318833839, Email tedysha@gmail.com

**II: Informed consent**

As to the information given ahead, participating in this study has no any risk. Your name will not be written on this form and the information you give will never be shared to others. You may not answer any questions that you do not want to answer and you may end this interview at any time you want. Now I would like to tell you that you are selected randomly to be participant of the study. Your genuine response to the questions was very important for the purpose of the study. At the same time we would like to appreciate your voluntarily participation in the interview after a thorough understanding of the information given to you. I have read this form or it has been read to me in the language I realized and understand all information stated above. Are you willing to participate in this study?

1. Yes 2. No

Signature of participants _______________ date ________/______/

**Status the study**: 1. Completed_____ 2. Refused______ 3. Participant absent_______ 4. Partially completed ______

Checked by supervisor: Name _______________________Signature_________ Date____/__/
