# Supplementary material for: Working in labor and delivery unit increases the odds of work place violence in Amhara region referral hospitals: Cross-sectional study
Source: PLoS One. 2021 Oct 20;16(10):e0254962. doi: 10.1371/journal.pone.0254962 (PMC8528509; doi:10.1371/journal.pone.0254962)
Supplement: S2 File — (DOCX) [file pone.0254962.s002.docx]

**Annex: English version of the Questionnaires**

**Instruction**: Please mark the number listed before the option to indicate your response and fill the black for open questions.

Code No _________

**Part I: Socio demographic and psychosocial information**

| Serial No | Questions | Response | Skip to Q |
| --- | --- | --- | --- |
| 101 | What is your age? | ___in year |  |
| 102 | Gender? | 1. male 2. female |  |
| 103 | What is your religion? | 1. 1.Orthodox 2. Muslim 3. 3.Protestant 4. 4.Catholic 5. 5. Others (Specify)….________ |  |
| 104 | What is your marital status? | 1. Single 2. Married 3. Divorced 4. Widowed |  |
| 105 | What is your educational level? | 1. Diploma 2. Degree 3. Masters 4. 4.Specialist   5. Resident |  |
| 106 | What is your profession? | 1. Nurse 2. Midwife 3. GP 4. IESO 5. Resident 6. Specialist |  |
| 107 | Ethnicity? | 1. Amhara 2. Tigre 3. 3. Oromo 4. Others specify |  |
| 108 | Work experience (in years)? | 1. 1 - 5 years 2. 6-10 years 3. 11-15 years 4. > 15 years |  |
| 109 | Monthly income in birr (including your over time) | ______________ birr |  |
| 110 | How do you rate yourself in communication skill? | 1. Very poor  2. Poor 3.  3.Good  4. Very good |  |
| 111 | How do you rate yourself in clinical skill? | 1. Very poor 2. Poor 3. Good 4. 4. Very good |  |
| 112 | How do you rate your work place satisfaction? | 1. Very dissatisfied 2. Dissatisfied 3. Satisfied 4. Very satisfied |  |

**Part II working unit and organization related questions**

| 201 | Where is your working unit? | 1. .ANC OPD 2. FP unit 3. GYN OPD 4. Labor deliver y ward 5. GYN inpatient 6. Others |  |
| --- | --- | --- | --- |
| 202 | Are you working in shifts? | 1. Yes 2. 2. No |  |
| 203 | If yes for Q 114 is that from 6pm to 7 am. | 1. Yes 2. No |  |
| 204 | Numbers of staffs in the unit? | 1. <5 2. 5-10 3. 11-15 4. >15 |  |
| 205 | Has your employer institution developed specific policies on work place violence? | 1. Yes 2. No 3. I don’t know |  |
| 206 | Do you know existing Measures to deal with violence when it happened? | 1. Yes 2. No 3. I don’t know |  |
| 207 | Is there a workplace violence dealing team when it happened in your hospital? | 1. Yes 2. No 3. . I don’t know |  |

**Part II: Workplace violence related questions**

| 301 | In the last 12 months, have you been physically attacked in your workplace? | 1. Yes 2. No |  |
| --- | --- | --- | --- |
| 302 | If yes for Q 301, please think of the last time that you were physically attacked and what type was it? | 1. Assault 2. Harassment 3. Physical trauma |  |
| 303 | In the last 12 months, have you been Intentionally verbally abused (emotional violence)in your workplace | 1. Yes 2. No |  |
| 304 | If yes, for Q 303, Please think of the last time that you were verbally attacked and what type was it? | 1. Threat verbal / written 2. Mobbing/bullying 3. Abuse 4. Disrespect 5. Repeated disturbance |  |
| 305 | In the last 12 months, have you been sexually harassed/ raped/ attempted rape in your workplace? | 1. Yes 2. No |  |
| 306 | In the last 12 months, have you been Intentionally Ethnically violated in your workplace? | 1. Yes 2. No |  |
| 307 | If yes for one of the above questions where did it happened | 1. At OPD 2. At ward 3. Staff rest room 4. Somewhere in compound 5. Others specify … |  |
| 308 | If yes for one of the above questions when do you faced for the last time? | 1. In the last 6 months 2. Before 6 months |  |
| 309 | If yes for questions above How often have you, been violated? | 1. All the time 2. Sometimes 3. Once |  |
| 310 | If yes for the above question Who Attacked you? | 1. Patient 2. Relative 3. Staff member 4. Management officials |  |
| 311 | If yes for question the above what was your reaction? | 1. Took no action 2. Told the person to stop 3. Report to senior staff 4. Help from association 5. Transferred 6. Completed incident form |  |
| 312 | Is there any action taken? | 1. Yes 2. No |  |
| 313 | What do you think the reason for violence? | 1. Long waiting time 2. Dissatisfaction from patient care and treatment 3. Misunderstanding 4. I don’t know 5. Others |  |
| 314 | If yes for Q No 028 who took action? | 1. Management/ employer 2. Syndicate 3. Community group 4. Police 5. Others |  |
| 315 | What was the Consequences to perpetrator/ abuser? | 1. None 2. Verbal warning issued 3. Care discontinued 4. Reported to police 5. Aggressor persecuted 6. Other |  |
| 316 | Incident could have been prevented. | 1. Yes 2. No |  |
| 317 | Have you given a support? | 1. Yes 2. No |  |
| 318 | If yes for Q 317, what types of support you get? | 1. Counseling offered 2. Compensation 3. Leave of a day’s 4. Others... |  |
| 319 | Are you satisfied with handling of incident? | 1. Very dissatisfied 2. Dissatisfied 3. Fare 4. Satisfied 5. Very Satisfied |  |
