# Supplementary material for: Working in labor and delivery unit increases the odds of work place violence in Amhara region referral hospitals: Cross-sectional study
Source: PLoS One. 2021 Oct 20;16(10):e0254962. doi: 10.1371/journal.pone.0254962 (PMC8528509; doi:10.1371/journal.pone.0254962)
Supplement: Attachment — Submitted filename: Response to Reviewers.docx [file pone.0254962.s003.docx]

**Author’s Point-by-Point Response to the reviewer’s and editors Reports**

**Title:** Working in labour and delivery increases the odds of work place violence

Eyaya Habtie: [eyuhabt143@gmil.com](mailto:eyuhabt143@gmil.com)

Abrham Walelign: [abrahawa2001@gmail.com](mailto:abrahawa2001@gmail.com)

Tigist Seid: [tigistseid@yahoo.com](mailto:tigistseid@yahoo.com)

Tewodros syum: [tedysha@gmail.com](mailto:tedysha@gmail.com)

Journal: PLOS ONE

Article type: Research article

**Point by point response to editors and reviewers**

First, the authors would like to thank PLOS ONE Research International academic editors and reviewers for reviewing our manuscript and providing the necessary comments to be corrected and re submitted. As per the comments are given, we have made corrections point by point to comment. The authors tried to answer all the issues raised by the reviewers.

**Reviewer 1**

Comment #1: “Factors related to violence can be classified into at least 4 domains. In addition to the three correctly proposed by the authors, we must not forget the perpetrators of violent acts”

Response: Thank you for your comment. As we revised, plenty articles that related to work place violence against health professionals working in obstetrics and gynaecology departments, majority of articles summarize in three categories. We were tried to incorporate articles with methodologically sound and convinced us. The other interesting point raised by you were subjects of perpetrators, in this research we were tried to study type 2 workplace violence (Perpetrator is a *customer* or *patient* or *employees* of the workplace). However, type 3 is includes former employee of the work place (e.g. recently fired employee assaults former supervisor) (see ref. No 32).

Comment #2: “Correlation between gender and the risks of exposure to violence”.

Response: Really, it is interesting view and we the authors considered.

Comment #3: “This research revealed that the prevalence of workplace violence was high. Government stakeholders and hospitals may give attention to reduce the burden of the problem for better health care in obstetrics, gynecology, and neonatal health. This can be address by drafting WPV incident reporting procedures, assign security personnel, making legislations and polices to make the health care place safe and conducive places both for patient and clients."

Response: A nice and valuable comment and moved as it recommend.

Comment #4: Enrich it with some more recent bibliographic sources.

Response: The authors considered a couple of research articles to be added. (I.e. ref. No 32and 33).

Comment #5: The References section is full of errors, it needs to be thoroughly checked and corrected:

Response: We thank you very much and it have been corrected

Finally we got the comments were as very valuable and constrictive and we are thank full for the reviewer.

**Reviewer 2:**

Comment #1: Throughout the manuscript there are many typos and unclear sentences; a linguistic revision is required.

Response: Thank you. We made a modification and took corrections on this part

Comment #2: The style of the references is inconsistent and some look incomplete:

Response: Thank you. We took action and modified

Comment #3: What were the variables taken into account to adjust the raw OR?

Response: We noticed and they are stated just before logistic regression table

Comment #4: A major concern regards adaptation of the questionnaire used for data collection:

Response: The tool was adapted from ILO/ICN/WHO/PSI; joint programme on workplace violence in the health sector workplace violence in the health sector country case studies research instruments survey questionnaire English version. In addition, some local published research articles done regarding work place violence against health professionals were revised and used. It was also commented by some research experts. Finally, it was validated by pretested and Cronbach’s alpha test was done, before the actual data was collected.

Comment #5: In table 3, please specify the years of training and differences between degree and diploma

Response: We made a modification on this part.

comment #6????
