# Supplementary material for: Working in labor and delivery unit increases the odds of work place violence in Amhara region referral hospitals: Cross-sectional study
Source: PLoS One. 2021 Oct 20;16(10):e0254962. doi: 10.1371/journal.pone.0254962 (PMC8528509; doi:10.1371/journal.pone.0254962)
Supplement: Attachment — Submitted filename: Response to Reviewers 22.docx [file pone.0254962.s004.docx]

**Author’s Point-by-Point Response to the reviewer’s and editors Reports**

**Title:** Working in labour and delivery increases the odds of work place violence

Eyaya Habtie: [eyuhabt143@gmil.com](mailto:eyuhabt143@gmil.com)

Abrham Walelign: [abrahawa2001@gmail.com](mailto:abrahawa2001@gmail.com)

Tigist Seid: [tigistseid@yahoo.com](mailto:tigistseid@yahoo.com)

Tewodros syum: [tedysha@gmail.com](mailto:tedysha@gmail.com)

Journal: PLOS ONE

Article type: Research article

**Point by point response to editors and reviewers**

First, the authors would like to thank PLOS ONE Research International academic editors and reviewers for reviewing our manuscript and providing the necessary comments to be corrected and re submitted. As per the comments are given, we have made corrections point by point to comment. The authors tried to answer all the issues raised by the reviewers and the editors.

**Editor’s comment**

Comment #1: “I suggest to removing the title in each chart because it is described by its caption”

Response: We thank very much to this interesting comment and we have removed the title after modifying the caption itself.

Comment #2: “Please review your reference list to insure that it is complete and correct. If you site papers that have been retracted please include the rationale …	”

Response: Thank you for your nice comment. As much as we can, we have made changes to on reference section as the PLOS ONE criteria and other journal standards). In addition, we would like to be clear that we did not cite retracted papers here.

**Reviewer’s comment**

Comment #1: “Organizational and working unit information" only two organizational aspects are presented: the Presence of policy for WPV and the Presence WPV dealing team. Explain that”.

Response: Really, it is interesting view. Under this section, these are the only factors or variable, which consistently used across literatures and listed in OSHA guideline. In addition some elements of the measurements regarding to organizational factors are not simple to evaluate for all referral hospitals.

Comment #2: “: I do not completely agree with the authors on this point, already highlighted in the previous review. The authors' response to the Reviewer #1's suggestion was incorrect because type III violence does not affect retired workers, but all workers, who exercise these forms of violence on their co-workers due to mobbing, bullying, lateral hostility, negative interactions. This article talks about both type II violence (as rightly stated) but also type III violence (the so-called worker on worker violence."

Response: A nice and valuable comment and modified as it recommend, because already the data was collected in the consideration of type II and type III. To be sure, it is possible to see on the questionnaire part.

Comment #3: “I also suggest replacing the reference supporting this classification by using the main source of this classification”

Response: Thank you for this valuable comment. We already considered it and modified.

Comment #5: The References section is full of errors, it needs to be thoroughly checked and corrected:

Response: We thank you very much and it have been addressed and corrected.

Finally we got the comments were as very valuable and constrictive and we are thank full for the reviewer.
